# Comparative analysis of selected methods for the assessment of antimicrobial and membrane-permeabilizing activity: a case study for lactoferricin derived peptides

**DOI:** 10.1186/1471-2180-8-196

**Published:** 2008-11-11

**Authors:** Susana Sánchez-Gómez, Marta Lamata, José Leiva, Sylvie E Blondelle, Roman Jerala, Jörg Andrä, Klaus Brandenburg, Karl Lohner, Ignacio Moriyón, Guillermo Martínez-de-Tejada

**Affiliations:** 1 Department of Microbiology and Parasitology, University of Navarra, 31080 Pamplona, Spain; 2 Clínica Universitaria, University of Navarra, 31080 Pamplona, Spain; 3 Torrey Pines Institute for Molecular Studies, San Diego, CA 92121, USA; 4 Laboratory of Biotechnology, National Institute of Chemistry, 1000 Ljubljana, Slovenia; 5 Forschungszentrum Borstel, Leibniz-Zentrum für Medizin und Biowissenschaften, D-23845 Borstel, Germany; 6 Institute of Biophysics and Nanosystems Research, Austrian Academy of Sciences, A-8042 Graz, Austria

## Abstract

**Background:**

Growing concerns about bacterial resistance to antibiotics have prompted the development of alternative therapies like those based on cationic antimicrobial peptides (APs). These compounds not only are bactericidal by themselves but also enhance the activity of antibiotics. Studies focused on the systematic characterization of APs are hampered by the lack of standard guidelines for testing these compounds. We investigated whether the information provided by methods commonly used for the biological characterization of APs is comparable, as it is often assumed. For this purpose, we determined the bacteriostatic, bactericidal, and permeability-increasing activity of synthetic peptides (n = 57; 9–13 amino acid residues in length) analogous to the lipopolysaccharide-binding region of human lactoferricin by a number of the most frequently used methods and carried out a comparative analysis.

**Results:**

While the minimum inhibitory concentration determined by an automated turbidimetry-based system (Bioscreen) or by conventional broth microdilution methods did not differ significantly, bactericidal activity measured under static conditions in a low-ionic strength solvent resulted in a vast overestimation of antimicrobial activity. Under these conditions the degree of antagonism between the peptides and the divalent cations differed greatly depending on the bacterial strain tested. In contrast, the bioactivity of peptides was not affected by the type of plasticware (polypropylene vs. polystyrene). Susceptibility testing of APs using cation adjusted Mueller-Hinton was the most stringent screening method, although it may overlook potentially interesting peptides. Permeability assays based on sensitization to hydrophobic antibiotics provided overall information analogous – though not quantitatively comparable- to that of tests based on the uptake of hydrophobic fluorescent probes.

**Conclusion:**

We demonstrate that subtle changes in methods for testing cationic peptides bring about marked differences in activity. Our results show that careful selection of the test strains for susceptibility testing and for screenings of antibiotic-sensitizing activity is of critical importance. A number of peptides proved to have potent permeability-increasing activity at subinhibitory concentrations and efficiently sensitized *Pseudomonas aeruginosa *both to hydrophilic and hydrophobic antibiotics.

## Background

Antimicrobial peptides (APs) participate in the first line of defense of a wide variety of organisms ranging from prokaryotes to mammals (for a review, see [1]). In humans, APs are essential components of innate immunity where they play key defensive and immunomodulatory roles [2]. To exert their antimicrobial action, APs have to bind first to their target cell membrane. The cationic nature and amphiphilic structure characteristic of the vast majority of APs enable them to interact specifically with negatively charged components of the microbial cell envelope (for reviews, see [3-5]). APs that are active on Gram-negative bacteria typically bind to lipopolysaccharide (LPS), a negatively charged molecule present in the outer membrane of these microorganisms [6].

Binding of APs to LPS causes the displacement of the divalent cations (e.g. Ca^2+ ^and Mg^2+^) that under physiological conditions stabilize the outer membrane via cross-bridging of neighboring LPS molecules. When APs act at concentrations higher or equal to their minimum inhibitory concentrations (MICs), they typically kill their target cells within minutes. At subinhibitory concentrations, APs alter the cell permeability barrier, thus making bacteria sensitive to agents that would be excluded by an intact outer membrane [7,8].

Deciding what type of assays is more appropriate to characterize the biological activity of APs *in vitro *is not straightforward. The literature reveals that sometimes researchers measure the bactericidal activity of APs under conditions that do not allow bacterial growth [9-11]. Others study the interaction between APs and their cell targets in buffers of varied ionic strength [12-15] or measure the MIC in culture media with non-standardized divalent cation content [16,17]. Moreover, for the calculation of the MIC by broth microdilution-based assays, some investigators emphasize the importance of using microplates made of polypropylene [18-20], whereas others perform their assays on automated turbidimetric systems [21-23], the latter being preferred by those dealing with high-throughput screening of antimicrobial compounds.

Methods used by researchers studying AP-dependent membrane permeabilization are even more diverse and include assays monitoring leakage of cells (i.e. ATP, enzymes; [24]), the uptake of various probes into the cell envelope [25,26] and indirect assays such as those measuring sensitization of the target cell to lysozyme [27] or to hydrophobic antibiotics [28].

Studies focused on the systematic characterization of APs are hampered by the lack of standard guidelines for testing these compounds. In the present report we investigated whether the information provided by methods commonly used for the biological characterization of APs is comparable, as it is often assumed. For this purpose, we determined the bacteriostatic, bactericidal, and permeability-increasing activity of synthetic peptides analogous to the LPS-binding region of human lactoferricin by a number of the most frequently used methods and carried out a comparative analysis. Our model organism was *Pseudomonas aeruginosa*, a Gram-negative opportunistic pathogen resistant to many antibiotics.

In the present work, we demonstrated that information provided by methods commonly used for the biological characterization of APs differ significantly depending upon changes in the test medium, growth conditions and bacterial species selected for the analysis.

## Results

### Characteristics of synthetic peptides

Synthetic peptides (n = 57) homologous to LF11, an 11 amino acid peptide analogous to the LPS-binding region of human lactoferricin [29], were designed to contain the following modifications: deletions, substitutions in the hydrophobic core, spacial constraint, elongation, charge increase, removal of charged amino acids and variation of amphipathicity. Representatives of these modifications are shown in Additional file 1.

The vast majority of the peptides were found to lack significant hemolytic activity and cytotoxicity when tested at 250 μg/mL on human red blood cells and HeLa cells, respectively (see Additional file 1).

### Comparison of methods used for susceptibility testing of antimicrobial peptides

All peptides were first screened for bactericidal activity under low ionic strength conditions on resting bacteria. For this purpose, we exposed bacterial cells in phosphate buffer (PB) to increasing concentrations of peptides and performed viable cell counts. Experimental designs similar to this are found in reports dealing with the biological characterization of peptides (see for instance [13]). These experiments were performed on *Escherichia coli*, *Pseudomonas aeruginosa *and *Bordetella bronchiseptica*, three Gram-negative pathogens differing in sensitivity to APs. As illustrated in Additional file 2, representatives of the main peptide classes exhibited a potent bactericidal activity on the three test organisms that was similar to that of polymyxin B (PMB).

The buffer used in the previous experiments was devoid of Ca^2+ ^and Mg^2+^, two divalent cations known to contribute to outer membrane stabilization. To study whether their presence had an effect on the susceptibility to peptides, we repeated the assays using PB supplemented with 1 mM Ca^2+ ^and 1 mM Mg^2+ ^(PB-CM medium). As shown in Additional file 2, these conditions lowered the bactericidal activity of almost all the peptides and this was observed in all bacterial strains. However, the magnitude of the decrease varied markedly depending upon the peptide and strain. Thus, whereas supplementation with Ca^2+ ^and Mg^2+ ^abrogated the activity of the peptides on both *B. bronchiseptica *and *P. aeruginosa*, such an effect was notably more modest in *E. coli*. In contrast, the activity of PMB was not significantly affected by the divalent cations.

To study the effect of using metabolically active bacteria in the assessment of antimicrobial activity, we determined the MIC and the minimum bactericidal concentrations (MBC) of several peptides according to Clinical and Laboratory Standards Institute (CLSI, formerly National Committee for Clinical Laboratory Standards) guidelines. For these assays, we used two different types of Mueller-Hinton (MH) medium differing in divalent cation concentration, namely, cation adjusted (containing 0.4–0.5 mM Mg^2+ ^plus 0.5–0.6 mM Ca^2+^), and non adjusted (containing 0.131–0.214 mM of Mg^2+ ^plus 0.07–0.14 mM Ca^2+^) provided by the same manufacturer. An increase in divalent cation concentration led to higher MICs in most cases and caused MBC rises of similar magnitude (2–4 fold) in *E. coli* and *B. bronchiseptica* (see Additional file 2). Regardless of cation concentration, metabolically active *P. aeruginosa *cells proved to be fully resistant to the peptides (see below).

Since the non-cation adjusted MH allowed ranking of the peptides by their MIC and MBC (see Additional file 2), we used this medium to test whether automated turbidimetry-based system and conventional methods yielded similar results. As shown in Additional file 3, both the conventional and the automated method led to similar MICs (i.e. twofold difference at most) for the majority of the peptides. Only in the case of peptide P50, when tested on *E. coli *and peptides P4 and P28 on *B. bronchiseptica*, a significant difference was detected in the MIC values of the two methods, although no method proved to be consistently more sensitive than the other. The growth of the *P. aeruginosa *strain was not inhibited even by the highest peptide concentration used, thereby hindering any meaningful comparison between the methods. In contrast, *B. bronchiseptica *was found to be much more sensitive and *E. coli *displayed an intermediate level of sensitivity. In almost all cases, peptides MBCs were almost identical to their corresponding MICs, thus indicating that the compounds are bactericidal at their MICs.

### Interference of plasticware in susceptibility assay

It has been reported that cationic peptides have affinity for certain plastics (i.e. polystyrene; [20]), and as a consequence some authors disfavor the use of microplates made of such material. To investigate this potential interference, we studied whether the composition of the microplate (polypropylene vs. polystyrene) affected the antibacterial activity of selected peptides differing in length, hydrophobicity and net charge when tested on *E. coli *ATCC 25922.

As shown in Additional file 4, the only peptide whose MIC improved in polypropylene plates was P13. However, the MBC of this compound was not significantly affected by the type of material used and peptide P11, which had an identical net charge and very similar primary structure (see Additional file 1), displayed the same MIC in both materials whereas its MBC was lower in polystyrene. Only one peptide (P46) improved its MBC when using polypropylene instead of polystyrene. In global terms, the composition of the microplate did not affect significantly the MIC or MBC assessed either on growing or on resting cells.

Of note, PMB had a higher activity (lower MIC value) when assayed in polypropylene but other lipopeptides such as C12LF11, a N-terminally acylated analogue of LF11 [29] displayed the opposite behavior (data not shown) indicating that acylation is not necessarily linked to affinity for polystyrene.

### Comparison of methods for measuring bacterial cell wall permeabilization

Since LPS plays a key role in outer membrane stability and all our peptides are analogous to the LPS-binding region of lactoferricin, we hypothesized that they could alter the outer membrane permeability of Gram-negative bacteria. Thus, we investigated whether some of the techniques used to measure bacterial permeabilization provide comparable information when applied to APs. As test organism, we used *P. aeruginosa*, which in preliminary experiments allowed discrimination between good and poor permeabilizers better than that of *E. coli*.

First, we studied the ability of the peptides to sensitize *P. aeruginosa *to novobiocin, a hydrophobic antibiotic that cannot reach its intracellular target (gyrase) due to its inability to cross an intact outer membrane. To quantify the synergistic effect, we calculated the ratio of novobiocin MICs in the absence (MIC ≥ 512 μg/mL) and in the presence of subinhibitory concentrations of the peptides. Specifically, for a combination to be considered synergistic MIC of each agent when combined have to be at least 4-times lower than that of the corresponding agent alone [30].

P14 was the most active compound since it caused a 4-fold decrease in novobiocin MIC when added at 6.25 μg/mL, a concentration at least 40 times lower than its own MIC (see Additional file 5). Interestingly, peptides with a highly charged C-terminus such as P10, and P15 also showed synergistic activity at low concentrations. Nevertheless, the most potent permeabilizer was peptide P22 which caused a 1024-fold drop in novobiocin MIC and also had the lowest MIC. However, this permeabilizing activity was only detectable at concentrations close to the peptide MIC.

To correct for the differences in peptide MICs, we calculated the Fractional Inhibitory Concentration (FIC) index (see the Methods section for details) for all the peptide-novobiocin combinations (see Additional file 5). Peptides with the lowest FIC indices (<0.2; i.e. the most potent permeabilizers) did not correspond necessarily to those having permeability-increasing activity (i.e. MIC ratios ≥ 2) at low concentrations. This was the case, for instance, for peptide P24. On the other hand, the best compounds as judged by their FIC indices, peptides P10 and P14, combined good permeabilizing activity at low concentration with a synergistic potential that progressively increased with peptide concentration. Similarly, P48 and P15 which were among the best permeabilizers based on MIC ratio also had low FIC indices.

In addition to this indirect method, we used a direct assay which measures the ability of peptides to promote the entry of a hydrophobic fluorescent probe (1-N-phenylnaphthylamine; NPN) into the cell membrane. This method identifies potent permeabilizers as those causing rapid NPN uptake leading to a high level of fluorescence. Preliminary experiments revealed that, to detect significant fluorescence increases, it was necessary to reach a peptide concentration in the cuvette of 50 μg/mL. Hence, this concentration was consistently used for the comparative analysis. As shown in Additional file 5, peptides causing the slowest and less prominent NPN-uptakes (i.e. P3, P28, P41 and P54) corresponded to those classified as poor permeabilizers by the novobiocin-sensitization technique. Interestingly, those combinations identified as synergistic by the latter method (at a peptide concentration of 50 μg/mL) also led to significant NPN rises, and peptides with high MIC ratios had the most potent NPN-uptake promoting activity. However, the direct (fluorescence-based) assay was clearly less discriminative than the indirect method as it failed to rank the permeability-increasing ability of peptides differing in up to 8 times MIC ratios (see for example peptides P8 and P15 when tested at 50 μg/mL).

### Peptide-mediated potentiation of antibiotics other than novobiocin

To study to what extent novobiocin-enhancing activity is valid as a criterion to screen for a broader permeabilizing ability of a given peptide, we combined peptide P22, a compound with a rather modest FIC index, with representative of antibiotic classes differing in mechanism of action. For these experiments we used the wild type *P. aeruginosa *strain PAO1, whose mechanisms of antibiotic resistance have been extensively studied [31]. As shown in Additional file 6, P22 formed synergistic combinations (FIC < 0.5) with all the antibiotics except for fusidic acid. This synergism was detected even at concentrations 20 times lower than the peptide MIC (31.25 μg/mL) in the case of rifampicin. Notably, subinhibitory concentrations of peptide P22 brought down to clinically useful levels the MIC of the majority of antibiotics including rifampicin (final MIC 0.07 μg/mL), erythromycin (final MIC 1 μg/mL), amoxicillin (final MIC 2 μg/mL), nalidixic acid (final MIC 2 μg/mL), ampicillin (final MIC 5 μg/mL) and novobiocin (final MIC 5 μg/mL). These compounds differed markedly in their octanol-water partition coefficient (see Additional file 6), suggesting that P22 is similarly useful at sensitizing *P. aeruginosa *to antibiotics regardless of their hydrophobicity. Results similar to these were obtained with peptides having FIC indices lower than P22 (data not shown) thus suggesting that the novobiocin-sensitization technique may serve to screen for global antibiotic-sensitizing activity.

Overexpression of multidrug efflux pumps such as MexAB-OprM confers *P. aeruginosa *with a high level of antibiotic resistance [32]. To study whether observations made with the wild type strain hold true when using strains with enhanced antibiotic resistance, we performed the antibiotic sensitization experiments using PAOLC1-6, an isogenic mutant of PAO1 carrying a mutation that results in overexpression of MexAB-OprM. As shown in Additional file 6, the PAOLC1-6 mutant displayed a level of susceptibility to antibiotics significantly lower than that of the wild type strain no matter the presence of P22. In fact, low peptide concentrations failed to sensitize the MexAB overexpressing mutant and even at the highest concentration P22 was unsuccessful at bringing the MIC of most antibiotics down to clinically useful levels.

## Discussion

When testing the activity of antimicrobial compounds in vitro it is important to use standardized conditions that allow meaningful assessments. In this way, the activity of a given compound against different microorganisms or of different compounds against the same microorganism can be compared. For this reason, there are accepted guidelines to measure the *in vitro *activity of classical antibiotics. Since guidelines for the susceptibility testing of cationic peptides are unavailable, we have compared methods commonly used for the analysis of antimicrobial and permeabilizing activity of APs.

A hallmark of APs is that their activity is antagonized by calcium and magnesium reflecting competition between divalent cations and APs for binding to LPS [3,8]. Interestingly, we found that the degree of antagonism between the peptides and the divalent cations in low-ionic strength medium greatly differed depending on the bacterial strain. Thus, addition of divalent cations counteracted in most of the cases bactericidal activity against *P. aeruginosa *and *B. bronchiseptica*, but did not confer protection on *E. coli*. This observation probably reflects that divalent cations more effectively crosslink *P. aeruginosa *and *B. bronchiseptica *LPS than their *E. coli *counterpart. Consistent with this hypothesis, *Pseudomonas *is known to be exquisitely sensitive to ethylenediaminetetraacetic acid (EDTA; [33]) and its outer membrane is more easily perturbed by chelators of divalent cations than that of *E. coli *[34]. These results clearly illustrate the importance of performing the susceptibility testing of novel peptides on several unrelated test strains differing in sensitivity to APs, as opposed to using only one organism.

We also found that testing under static conditions in low-ionic strength solvents results in a vast overestimation of bactericidal activity and precludes discrimination among peptides. Furthermore, these conditions are probably far from the physiological environment in the human body. It has been reported that most body fluids, including sputum, airway surface liquid, and serum/plasma contain concentrations of divalent cations between 1 and 2 mM [35]. In fact, addition of 1 mM Mg^2+ ^and Ca^2+ ^to our low-ionic strength medium sufficed in many cases to abolish the peptide bactericidal activity. Moreover, cells maintained in buffers lacking any carbon source cannot deploy energy-dependent defense mechanisms, such as those efflux systems that can pump cationic peptides out of the cells [36]. Likewise, the activities of several types of APs have been shown to be influenced by transmembrane potential which in turn requires active cell metabolism [3]. Nevertheless, testing under low-ionic strength conditions may be useful to increase the level of sensitivity in an initial screening of bactericidal activity as previously shown [37].

Our study indicates that susceptibility testing of APs using cation adjusted Mueller-Hinton is probably the most stringent screening method. However, as we showed, this method may overlook some potentially interesting peptides such as compounds intended to be used not as antimicrobials but as antibiotic-sensitizing agents. Indeed, a way to increase the sensitivity threshold and hence the discriminative potential of this type of screenings is to use non-cation adjusted Mueller-Hinton as test medium.

It has been claimed that cationic peptides of disparate nature bind polystyrene [19] whereas other authors reported binding of nisin and enterocin to polypropylene [38]. Even though peptides selected for our analysis differed up to three times in hydrophobicity and up to twice in net-charge, we obtained similar results in polystyrene and polypropylene. Therefore, it appears that such factor is not relevant for all polycations. In fact, none of the studies reporting affinity of several classes of cationic peptides for plasticware (e.g. [20,39,40]) include lactoferricin derived peptides, the type of compounds used in the present work.

Concerning permeability assays, our results are consistent with the notion that good permeabilizers do not necessarily correspond to compounds with potent bactericidal activity. This has been shown to be the case with the enzymatic derivative of PMB, PMB nonapeptide, whose antimicrobial activity is markedly reduced with respect to PMB while, as a permeabilizer, it retains the potency of its parent molecule ([41], and our own unpublished results). Observations similar to ours have been reported by others using agents of disparate nature including spermidine and other polyamines ([42,43], cholic acid derivatives [44] as well as cationic steroid antibiotics [45].

In addition, we demonstrated that methods measuring the ability of peptides to induce bacterial sensitization to hydrophobic antibiotics such as novobiocin provide information that is overall analogous – though not quantitatively comparable- to that of tests based on the uptake of hydrophobic fluorescent probes (e.g. NPN). We also showed that the former have several advantages over the latter including the possibility of calculating useful quantitative parameters such as the FIC index and the MIC ratio, as well as better discriminative potential and ease of performance. In turn, fluorescence-based techniques provide rapid results and thus can be used to identify the most promising candidate compounds in initial screenings of synergistic activity.

Finally, careful selection of the test strain for screenings of antibiotic-sensitizing activity is of key importance, as our results show. Thus, most of the peptides permeabilized *E. coli *outer membrane (data not shown), whereas only the most potent permeabilizers were active on wild type *P. aeruginosa*. However, even these compounds were barely synergistic when tested on a mutant strain overexpressing the MexAB-OprM efflux system, a phenotype very frequent in clinical isolates of *P. aeruginosa *[46].

In summary, we tested an array of related peptides using a range of methods that allowed us to best characterize their biological activity. By using these methods, we identified peptides that although not highly bactericidal under stringent conditions, were endowed with potent permeability-increasing activity at subinhibitory concentrations. We also found that some of them efficiently sensitized *P. aeruginosa *not only to hydrophobic antibiotics, as already reported for other cationic compounds [44,45,47-50], but also to hydrophilic ones.

## Conclusion

Although testing under static conditions in low-ionic strength solvents may be useful to increase the level of sensitivity in screenings of bactericidal activity, it overestimates such activity and precludes discrimination among peptides. In contrast, cation adjusted Mueller-Hinton is the most stringent screening medium but it may overlook potentially interesting peptides. The use of non-cation adjusted Mueller-Hinton offers a good compromise between sensitivity and discriminative potential. Regardless the testing conditions, the composition of the microplate (polypropylene vs. polystyrene) did not affect the peptides antibacterial activity.

Permeability assays based on sensitization to hydrophobic antibiotics provide overall information analogous – though not quantitatively comparable- to that of tests based on the uptake of hydrophobic fluorescent probes. For screenings of permeabilizing and antibacterial activity, careful selection of the test strains is critical since it greatly affects the conclusions of the study.

A number of peptides proved to have potent permeability-increasing activity at subinhibitory concentrations and efficiently sensitized *Pseudomonas aeruginosa *both to hydrophilic and hydrophobic antibiotics.

## Methods

### Bacterial strains and culture conditions

*Bordetella bronchiseptica *11844-99 and *Pseudomonas aeruginosa *4158-02 were isolated at the *Clínica Universitaria de Navarra *(University Hospital of Navarra) from respiratory samples taken from patients suffering bronchiectasis and a nosocomial respiratory infection, respectively. The quality control collection strains *Escherichia coli *ATCC 25922, *P. aeruginosa *ATCC 27853 and *S. aureus *ATCC 25923 were used throughout the study to monitor antibiotic activity. Strain PAOLC1-6, the MexAB-OprM overproducing mutant of *P. aeruginosa *PAO1, has already been described [51] and was a kind gift of Drs. M. Carmen Conejo, Luis Martínez-Martínez and Alvaro Pascual (University of Seville, Seville, Spain). All strains were routinely grown at 37°C in LB (Luria Bertani; Pronadisa, Madrid, Spain) broth or LB agar plates (LB supplemented with 1.5% agar (Pronadisa)).

### Peptides

Peptides were synthesized with an amidated C-terminus at the Torrey Pines Institute for Molecular Studies (San Diego, CA, USA) using tBoc (tert-butyloxycarbonyl)) solid phase chemistry and purified by RP-HPLC. Mass spectroscopy analysis verified that their purity was 75%. Polymyxin B (PMB) and its deacylated derivative PMB nonapeptide (PMBN) were purchased from Sigma-Aldrich (St. Louis, MO, USA).

### Antimicrobial susceptibility testing

#### Broth microdilution assay

The peptides MICs were determined in Mueller-Hinton (MH) medium (Difco Laboratories, Sparks, MD, USA) by the broth microdilution assay following recommendations of the Clinical and Laboratory Standards Institute [52]. Briefly, serial twofold dilutions of the peptides were made in MH broth and dispensed into 96-well U-bottom polystyrene microtiter plates (TPP, Trasadingen, Switzerland). Bacterial cells grown for 18–20 h on agar plates were suspended in saline and the turbidity was adjusted to an O.D_600 _of 0.04 (1 × 10^7 ^CFU/mL, approximately). A hundred-fold dilution of this suspension prepared in MH broth was mixed 1:1 with 100 μL of each of the corresponding peptide dilution. Plates were incubated at 37°C without shaking for 18–20 h and the MIC was determined visually. Minimum bactericidal concentration (MBC) was determined by plating aliquots from those wells with no growth onto MH agar plates. MBC was defined as the lowest concentration of the peptide causing a 99.9% loss of viability with respect to the CFU/mL inoculated.

#### Bioscreen assay

MICs were also determined by an automated turbidimetric-based system (Bioscreen C, Labsystem, Helsinki, Finland), which measures absorbance of the culture at regular intervals. Assays were performed in MH broth using Bioscreen polystyrene honeycomb 100-well plates. Inocula were prepared and mixed with the test peptides as indicated above for the CLSI-based MIC assay. Cell suspensions were grown at 37°C with shaking (control set at "medium r.p.m." position) and MIC was considered as the minimum concentration of peptide that inhibited any increment of turbidity after 24 h.

#### Assays in media differing in ionic strength

The activity of the peptides was determined on non-growing bacteria by incubating bacterial suspensions containing number of CFU/mL identical to those used for the MIC assay (5 × 10^4 ^per microplate well) in low-ionic strength medium (20 mM phosphate, pH 7.0) with or without 1 mM Ca ^2+ ^and 1 mM Mg ^2+^. After 18 hours, aliquots were plated onto MH agar to determine the presence of viable bacteria, as described above for the standard MBC assay.

#### Assays in polypropylene vs. polystyrene labware

The peptides MICs were also determined using polypropylene labware by the conventional broth microdilution assay as described above. For these experiments, polypropylene 96 well round bottom plates (Greiner; Sigma-Aldrich, USA) were used. Both this and all the previous antimicrobial susceptibility assays were performed at least in duplicate.

### Hemolytic activity and cytotoxicity

The peptides hemolytic activities were determined using human erythrocytes (RBCs) in 96-well tissue culture plates. One hundred μL of a 0.5% RBC suspension was added to an equal volume of a 500 μg/mL peptide dilution. After 1 h of incubation at 37°C, plates were centrifuged at 2,800 rpm (1,420 × g) for 5 min and the optical density of the supernatant was measured at 414 nm. Values for 0% and 100% of lysis were obtained by supplementing RBCs with PBS and Triton X-100 (1%; final concentration), respectively. All assays were performed in duplicate and the values of percent lysis were within a 2% standard deviation range.

### Synergy testing

The ability of the peptides to induce bacterial sensitization to antibiotics was determined by a standard checkerboard titration method in 96-well polystyrene microtiter plates [30]. For this purpose, serial dilutions of the two antimicrobial agents were mixed together in a microtiter plate so that each row contained a fixed amount of peptide and increasing amounts of the antibiotic. Inocula and growth conditions were identical to those described for MIC determination. To assess the synergy between antibiotics and peptides, the Fractional Inhibitory Concentration (FIC) index of each combination was calculated according to the following formula: [(A)/MIC_A_] + [(P)/MIC_P_] = FIC_A _+ FIC_P _= FIC index, where MIC_A _and MIC_P _are the MICs of the antibiotic and peptide determined separately, and (A) and (P) are the MICs of the antibiotic and peptide when determined in combination. A given peptide-antibiotic combination was considered as synergistic if its FIC index was ≤ 0.5. Peptides with a MIC higher than the maximum concentration tested (250 μg/mL) were arbitrarily assigned a MIC value of 500 μg/mL.

### Permeabilization assays

The peptides permeabilizing activity on the bacterial cell envelope was assessed by quantifying the uptake of 1-N-phenylnaphthylamine (NPN). These experiments were performed essentially as described by Loh and collaborators [25] with some modifications. Briefly, bacterial cells were grown in LB broth to mid-logarithmic phase, washed in 5 mM HEPES buffer (N-2-hydroxyethylpiperazine-N'-2-ethanesulfonic acid, pH 7.2) and resuspended at an OD_600 _of 0.5 in HEPES supplemented with 0.1% glucose. Freshly prepared bacterial suspensions were allowed to stabilize at 37°C for 10 min in quartz cuvettes. NPN was added at a final concentration of 10 μM and fluorescence was measured with a fluorometer (LS-50, Perkin-Elmer, Wellesley, MA, USA) using the time drive recording mode, slit widths of 2.5 nm and excitation and emission wavelengths of 350 nm and 420 nm, respectively. Peptides were added to the cuvette at a final concentration of 50 μg/mL.

## Authors' contributions

KL, RJ, SB, JA and KB designed the peptide sequences and conceived of the study. SB characterized the peptides and performed the toxicity analyses. SS and ML designed and carried out most of the experimental work under the supervision of JL, GMT and IM. GMT and IM edited the manuscript. All authors read and approved the final manuscript.

## Supplementary Material

Additional file 1 **Characteristics of representative synthetic peptides used in this study.** The sequence of the peptides as well as their hemolytic and cytotoxic activity are provided. Click here for file

Additional file 2 **Influence of ionic strength and concentration of cations on the antibacterial activity of LF11 derivatives.** the bacteriostatic and bactericidal activity of the peptides measured in media differing in ionic strength and cation concentration are provided. Click here for file

Additional file 3 **Antibacterial activity of LF11 derivatives assessed by a conventional microbroth assay and by the Bioscreen system.** comparison of the antibacterial activity of the peptides measured by conventional microbroth assay and by an automated turbidimetric system. Click here for file

Additional file 4 **Influence of plastics on the activity of peptides against *E. coli *ATCC 25922.** Comparison of the antibacterial activity of the peptides measured either in polypropylene or in polystyrene plasticware by two different methods. Click here for file

Additional file 5 **Permeabilizing activity of peptides added at subinhibitory concentrations on *Pseudomonas aeruginosa *4158-02.** comparison of the permeability-increasing activity of the peptides measured by two different methods. Click here for file

Additional file 6 **Permeabilizing activity of peptide P22 on *Pseudomonas aeruginosa *wild type and its isogenic multidrug efflux pump.** quantification of the ability of the peptides to sensitize *Pseudomonas aeruginosa *to antibiotics and influence of efflux pump expression in antibiotic susceptibility Click here for file
