# Supplementary material for: Comparative analysis of selected methods for the assessment of antimicrobial and membrane-permeabilizing activity: a case study for lactoferricin derived peptides
Source: BMC Microbiol. 2008 Nov 11;8:196. doi: 10.1186/1471-2180-8-196 (PMC2615442; doi:10.1186/1471-2180-8-196)
Supplement: Additional file 1 — Characteristics of representative synthetic peptides used in this study. The sequence of the peptides as well as their hemolytic and cytotoxic activity are provided. [file 1471-2180-8-196-S1.pdf]

Table 1. Characteristics of representative synthetic peptides used in this study

PEPTIDE	SEQUENCE	RELEVANT FEATURES	HEMOLYSIS [2]	CYTOTOXICITY [3]
**Parent peptide**				
**LF11**	FQWQRNIRKVR-NH_2	Derived from human lactoferricin (see reference [29])		
**LF11 derived [1]**				
**P 2**	FWQRIRKVR-NH_2	Internal single amino acid deletions	1	83
**P 3**	FWRNIRKVR-NH_2		0	82
**P 4**	FWQRNIRVR-NH_2		5	101
**P 8**	FWQRNIRKVKK-NH_2		3	103
**P 10**	FWQRNIRKVKKK-NH_2		3	96
**P 11**	FWQRNIRKVRRRR-NH_2	Positively charged C-terminal extension	2	111
**P 13**	FWQRNIRKVKKKK-NH_2		0	109
**P 14**	FWQRNIRKVKKKI-NH_2		6	110
**P 15**	FWQRRIRKVRR-NH_2		4	108
**P 17**	RFWQRNIRKVRR-NH_2		1	108
**P 21**	RFWQRNIRKYR-NH_2	Positively charged N and C terminal extensions	0	93
**P 22**	RFWQRNIRKYRR-NH_2		0	91
**P 24**	RRWQRNIRKYRR-NH_2		0	99
**P 25**	cFWQRNIRKVRc-NH_2	Disulfide insertion; Cyclic	5	97
**P 28**	CFWQRNIRKVRC-NH_2	Disulfide insertion; Linear	5	101
**P 34**	FYQRNIRKVR-NH_2		8	107
**P 36**	FWQRNIRIRR-NH_2	Single hydrophobic substitutions	0	114
**P 40**	FWQRNIFKVR-NH_2		0	115
**P 41**	FWQRNIYKVR-NH_2		0	103
**P 43**	FIWQRNIRKVR-NH_2	Substitution of Q in position 2	0	97
**P 44**	FLWQRNIRKVR-NH_2		2	99
**P 46**	FWARNIRKVR-NH_2	substitution of Q in position 4	0	92
**P 48**	FWLRNIRKVR-NH_2		0	99
**P 49**	FWVRNIRKVR-NH_2		0	103
**P 50**	FWPRNIRKVR-NH_2		0	91
**P 54**	FWQRNISKVR-NH_2	substitution R for S in position 8	10	102
**P 55**	FWQRNIRSVR-NH_2	substitution K for S in position 9	8	90

[1] Modifications were selected based on the tertiary structure of LF11 and SAR analysis (to be published elsewhere)
[2] Measured at a peptide concentration of 250 (μg/mL) and expressed as percentage of red blood lysis
[3] Measured at a peptide concentration of 250 (μg/mL) and expressed as percentage of HeLa cell survival
