# Supplementary material for: Comparative analysis of selected methods for the assessment of antimicrobial and membrane-permeabilizing activity: a case study for lactoferricin derived peptides
Source: BMC Microbiol. 2008 Nov 11;8:196. doi: 10.1186/1471-2180-8-196 (PMC2615442; doi:10.1186/1471-2180-8-196)
Supplement: Additional file 2 — Influence of ionic strength and concentration of cations on the antibacterial activity of LF11 derivatives. the bacteriostatic and bactericidal activity of the peptides measured in media differing in ionic strength and cation concentration are provided. [file 1471-2180-8-196-S2.pdf]

Table 2. Influence of ionic strength and concentration of cations on the antibacterial activity of LF11 derivatives

	STATIC CONDITIONS						ACTIVE GROWTH CONDITIONS							
	E. coli ATCC 25922		*B. bronchiseptica* 11844-99		*P. aeruginosa* 4158 – 02		*E. coli* ATCC 25922				*B. bronchiseptica 11844-99 (CUN)*			
							Non-cation adjusted[3]		Cation adjusted[4]		Non-cation adjusted		Cation adjusted	
**PEPTIDE**	**PB[1]**	**PB-CM[2]**	**PB**	**PB-CM**	**PB**	**PB-CM**	**MIC[5]**	**MBC[6]**	**MIC**	**MBC**	**MIC**	**MBC**	**MIC**	**MBC**
**LF11**	2	256	8	>256	4	>256	256	>256	>256	>256	256	256	>256	>256
**P14**	0.5	8	8	>256	2	> 256	>256	>256	>256	>256	8	8	16	32
**P15**	0.5	8	8	64	2	> 256	32	32	64	64	16	32	32	64
**P21**	1	2	0.5	>256	0.5	> 256	128	128	128	256	32	128	128	256
**P22**	0.5	2	2	>256	2	> 256	64	64	126	256	32	32	16	256
**P24**	1	8	16	>256	1	> 256	256	256	256	>256	256	256	256	256
**P48**	0.5	32	2	>256	2	> 256	64	64	256	>256	8	16	16	16
**P49**	1	8	1	>256	0.5	> 256	64	64	128	>256	32	> 256	64	128
**P50**	1	4	2	>256	0.5	> 256	32	32	128	256	16	16	64	128
**P55**	1	32	4	>256	4	> 256	128	128	128	>256	128	128	256	>256
**PMB**	1	0.5	0.5	0.5	1	1	1	1	1	1	1	1	0.5	0.5

[1] PB: minimum bactericidal concentration in μg/mL determined by exposing a bacterial suspension to the peptide for 18 h in phosphate buffer 20 mM; pH= 7.0

[2] PB-CM: determined as in 1 but PB was supplemented with 1mM Ca^{++} and 1mM Mg^{++}

[3] Mueller Hinton broth culture containing approximately 0.131-0.214 mM of Mg^{2+} and 0.07-0.14 mM Ca^{2+} as provided by the manufacturer

[4] Mueller Hinton II broth culture containing 0.4-0.5 mM of Mg^{2+} and 0.5-0.6 mM Ca^{2+} as provided by the manufacturer

[5] MIC: minimum inhibitory concentration in μg/mL determined by a conventional microbroth-based assay

[6] MBC: minimum bactericidal concentration in μg/mL determined by plating aliquots of non-cloudy wells from the conventional microbroth-based MIC assay
