# Supplementary material for: Comparative analysis of selected methods for the assessment of antimicrobial and membrane-permeabilizing activity: a case study for lactoferricin derived peptides
Source: BMC Microbiol. 2008 Nov 11;8:196. doi: 10.1186/1471-2180-8-196 (PMC2615442; doi:10.1186/1471-2180-8-196)
Supplement: Additional file 3 — Antibacterial activity of LF11 derivatives assessed by a conventional microbroth assay and by the Bioscreen system. comparison of the antibacterial activity of the peptides measured by conventional microbroth assay and by an automated turbidimetric system. [file 1471-2180-8-196-S3.pdf]

Table 3. Antibacterial activity of LF11 derivatives assessed by a conventional microbroth assay and by the Bioscreen system

**PEPTIDE**	***E. coli* ATCC25922**			***P. aeruginosa* 4158 – 02**			***B. bronchiseptica* 11844-99**		
	**MIC[1]**	**MIC-B[2]**	**MBC[3]**	**MIC**	**MIC-B**	**MBC**	**MIC**	**MIC-B**	**MBC**
**LF11**	256	>256	>256	>256	>256	>256	256	256	256
**P 4**	64	64	64	> 256	>256	> 256	64	256	64
**P 11**	32	64	32	256	256	256	32	32	32
**P 21**	128	128	128	> 256	>256	> 256	32	nt	128
**P 28**	> 256	>256	> 256	> 256	>256	> 256	128	4	128
**P 40**	64	128	64	> 256	>256	> 256	16	32	16
**P 44**	128	128	128	> 256	>256	> 256	16	32	16
**P 49**	64	64	64	256	>256	>256	32	64	> 256
**P 50**	32	256	32	> 256	>256	> 256	16	16	16
**P 55**	128	256	128	> 256	>256	> 256	128	128	128
**PMB**	1	1	1	1	1	1	≤ 0.5	≤ 0.5	≤ 0.5

[1] MIC: minimum inhibitory concentration in µg/mL determined by a conventional microbroth-based assay in non-cation adjusted Mueller Hinton medium
[2] MIC-B: minimum inhibitory concentration in µg/mL determined by the automated Bioscreen system in non-cation adjusted Mueller Hinton medium
[3] MBC: minimum bactericidal concentration in µg/mL determined by plating aliquots of non-cloudy wells from the conventional microbroth-based MIC assay
