# Supplementary material for: Comparative analysis of selected methods for the assessment of antimicrobial and membrane-permeabilizing activity: a case study for lactoferricin derived peptides
Source: BMC Microbiol. 2008 Nov 11;8:196. doi: 10.1186/1471-2180-8-196 (PMC2615442; doi:10.1186/1471-2180-8-196)
Supplement: Additional file 4 — Influence of plastics on the activity of peptides against E. coli ATCC 25922. Comparison of the antibacterial activity of the peptides measured either in polypropylene or in polystyrene plasticware by two different methods. [file 1471-2180-8-196-S4.pdf]

Table 4. Influence of plastics on the activity of peptides against *E. coli* ATCC 25922

Peptide	n[1]	GRAVY[2]	Q[3]	POLYSTYRENE			POLYPROPYLENE		
				MIC[4]	MBC[5]	PB[6]	MIC	MBC	PB
P 3	9	-1.144	5	> 256	> 256	2	256	256	2
P 4	9	-1.100	4	32	32	4	32	64	4
P 11	13	-2.100	8	32	32	2	32	128	2
P 13	13	-1.962	8	128	128	2	32	256	2
P 17	12	-1.900	7	256	256	2	128	256	4
P 21	11	-1.664	6	64	64	2	32	64	1
P 25	12	-0.733	5	64	256	2	32	256	2
P 28	12	-0.733	5	> 256	> 256	1	> 256	> 256	2
P 34	10	-1.420	5	> 256	> 256	2	> 256	> 256	4
P 40	10	-0.650	4	64	128	2	32	64	1
P 43	11	-0.850	5	32	64	8	32	32	8
P 44	11	-0.909	5	128	128	2	128	128	1
P 46	10	-0.850	5	32	256	2	32	64	1
P 49	10	-0.610	5	32	32	2	32	32	1
P 50	10	-1.190	5	32	64	2	32	32	2
P 55	10	-1.070	4	128	128	4	128	256	2
PMB[7]	10	-1.820	5	0.5	0.5	0.5	< 0.125	0.5	0.5

[1] Number of amino acid residues

[2] GRAVY index: Grand average of hydropathicity (the more positive the score, the more hydrophobic the overall sequence)

[3] Q: net charge

[4] MIC: minimum inhibitory concentration in μg/mL determined by a microbroth-based assay in non-cation adjusted Mueller-Hinton medium

[5] MBC: minimum bactericidal concentration in μg/mL determined by plating aliquots of non-cloudy wells from the microbroth-based MIC assay

[6] PB: minimum bactericidal concentration in μg/mL determined by exposing a bacterial suspension to the peptide for 18 h in phosphate buffer 20 mM; pH= 7.0

[7] Diaminobutyric acid residues present in PMB were substituted for lysine residues to calculate the GRAVY index of the peptidic moiety of PMB.
