# Supplementary material for: Comparative analysis of selected methods for the assessment of antimicrobial and membrane-permeabilizing activity: a case study for lactoferricin derived peptides
Source: BMC Microbiol. 2008 Nov 11;8:196. doi: 10.1186/1471-2180-8-196 (PMC2615442; doi:10.1186/1471-2180-8-196)
Supplement: Additional file 5 — Permeabilizing activity of peptides added at subinhibitory concentrations on Pseudomonas aeruginosa 4158-02. comparison of the permeability-increasing activity of the peptides measured by two different methods. [file 1471-2180-8-196-S5.pdf]

**Table 5.** Permeabilizing activity of peptides added at subinhibitory concentrations on *Pseudomonas aeruginosa* 4158-02

| | | MIC RATIO of novobiocin at the peptide concentration (µg/mL) indicated[2] | | | | | | | |
|---|---|---|---|---|---|---|---|---|---|
| **PEPTIDE** | **MIC (µg/mL)** [1] | 0.78 | 3.125 | 6.25 | 12.5 | 25 | 50 | **FIC** [3] **index** | **NPN uptake** [4] |
| P2 | 250 | < 2 | < 2 | < 2 | 2 | 4 | 16 | 0.263 | 145 ± 34 |
| P3 | >250 | < 2 | < 2 | < 2 | < 2 | < 2 | 2 | > 0.5 | 20 ± 5 |
| P8 | >250 | < 2 | < 2 | < 2 | 2 | 4 | 8 | 0.225 | 247 ± 7 |
| P10 | >250 | < 2 | < 2 | 2 | 4 | 16 | 16 | 0.113 | 224 ± 23 |
| P14 | >250 | < 2 | < 2 | 4 | 8 | 16 | 32 | 0.113 | 336 ± 30 |
| P15 | 250 | < 2 | < 2 | 2 | 4 | 16 | 64 | 0.163 | 280 ± 30 |
| P22 | 31.25 | < 2 | < 2 | < 2 | 32 | 1024 | n.f. [5] | 0.431 | 237 ± 25 |
| P24 | >250 | < 2 | < 2 | < 2 | < 2 | 8 | 32 | 0.131 | 272 ± 37 |
| P28 | >250 | < 2 | < 2 | < 2 | < 2 | < 2 | < 2 | > 0.5 | 50 (110) |
| P36 | 250 | < 2 | < 2 | < 2 | < 2 | < 2 | 4 | 0.450 | 253 ± 73 |
| P41 | >250 | < 2 | < 2 | < 2 | < 2 | < 2 | < 2 | > 0.5 | 35 (105) |
| P48 | 250 | < 2 | < 2 | < 2 | 8 | 32 | 256 | 0.131 | 287 ± 25 |
| P54 | >250 | < 2 | < 2 | < 2 | < 2 | < 2 | < 2 | > 0.5 | 20 (120) |
| PMBN | 31.25 | 128 | 32 | 32 | 32 | n.f. | n.f. | 0.033 | 359 ± 32 |

[1] Minimum inhibitory concentration of the peptides determined by a conventional microbroth-based assay in non-cation adjusted Mueller Hinton medium

[2] Ratio of novobiocin MICs in the absence and in the presence of the peptide. Novobiocin MIC was equal or higher than 512 µg/mL in the absence of peptide

[3] Fractional inhibitory concentration index (see Material and Methods section for details)

[4] Net increment of fluorescence after addition of 1-N-phenylnaphthylamine and subsequent (10 s, approximately) stabilization. The final peptide concentration was 50 µg/mL For those peptides that induced a gradual incorporation of the probe, value in parenthesis corresponds to reading after 120 s.

[5] not feasible (peptide inhibits growth by itself at that concentration)
