# Supplementary material for: Comparative analysis of selected methods for the assessment of antimicrobial and membrane-permeabilizing activity: a case study for lactoferricin derived peptides
Source: BMC Microbiol. 2008 Nov 11;8:196. doi: 10.1186/1471-2180-8-196 (PMC2615442; doi:10.1186/1471-2180-8-196)
Supplement: Additional file 6 — Permeabilizing activity of peptide P22 on Pseudomonas aeruginosa wild type and its isogenic multidrug efflux pump. quantification of the ability of the peptides to sensitize Pseudomonas aeruginosa to antibiotics and influence of efflux pump expression in antibiotic susceptibility [file 1471-2180-8-196-S6.pdf]

Table 6. Permeabilizing activity of peptide P22 on *Pseudomonas aeruginosa* wild type and its isogenic multidrug efflux pump overexpressing mutant

				**MIC RATIO of antibiotic at the concentration of peptide P22 (μg/mL) indicated [3]**					
**STRAIN**	**ANTIBIOTIC**	**Log*P* [1]**	**MIC (μg/mL) [2]**	**1,56**	**3,125**	**6,25**	**12,5**	**25**	**FIC index [4]**
PAO1 (wt)	CHLORAMPHENICOL	1.14	256	< 2	2	4	8	8	0,45
	FUSIDIC ACID	6.75	1024	< 2	< 2	2	8	32	0,52
	NALIDIXIC ACID	1.59	512	< 2	4	4	16	256	0,35
	AMOXICILLIN	0.87	2048	< 2	< 2	16	16	1024	0,26
	AMPICILLIN	1.35	1024	< 2	2	2	32	256	0,43
	ERYTHROMYCIN	3.06	128	< 2	2	4	8	128	0,45
	RIFAMPICIN	4.24	8	2	4	4	12	108	0,35
PAOLC1-6 (*nalB*)	CHLORAMPHENICOL	1.14	256	< 2	< 2	4	8	16	0,45
	FUSIDIC ACID	6.75	4096	< 2	< 2	< 2	32	64	0,43
	NALIDIXIC ACID	1.59	1024	< 2	< 2	4	8	64	0,45
	CEFOTAXIME	0.64	64	< 2	< 2	2	2	8	0,7
	TETRACYCLINE	-3.6	32	< 2	< 2	2	4	8	0,65
	TICARCILLIN	1	64	< 2	< 2	2	4	16	0,65

[1] Log octanol-water partition coefficient (taken from ChemIDplus Advanced; see http://chem.sis.nlm.nih.gov/chemidplus)

[2] Minimum inhibitory concentration of the antibiotic determined by a microbroth-based assay in non-cation adjusted Mueller-Hinton medium

[3] Ratio of antibiotic MICs (determined as in 2) in the absence and in the presence of peptide P22 at the indicated concentration. MIC of P22 alone= 31.25 μg/mL

[4] Fractional inhibitory concentration index (see Material and Methods section for details)
